# Surface Condition Evolution and Fatigue Evaluation after Different Surface Processes for TiAl_47_Cr_2_Nb_2_ Alloy

**DOI:** 10.3390/ma15165491

**Published:** 2022-08-10

**Authors:** Wen Yu, Yajun Yin, Jianxin Zhou, Qian Xu, Xin Feng, Hai Nan, Jiabin Zuo, Xiangning Wang, Xianfei Ding

**Affiliations:** 1 State Key Laboratory of Materials Processing and Die and Mould Technology, Huazhong University of Science and Technology, Wuhan 430074, China; 2 Cast Titanium Alloy R & D Center, Beijing Institute of Aeronautical Materials, Beijing 100095, China; 3 Beijing Engineering Research Center of Advanced Titanium Alloy Precision Forming Technology, Beijing 100095, China; 4 Beijing Institute of Aeronautical Materials Co., Ltd., Beijing 100094, China

**Keywords:** TiAl alloy, surface condition, fatigue behaviors, investment casting

## Abstract

The TiAl_47_Cr_2_Nb_2_ alloy fatigue specimens were prepared by investment casting, and three kinds of surface processes were applied to fatigue specimens. These three processes were sand-blasting (SB), sand-blasting and shot-peening (SBSP) and sand-blasting and mechanical grinding (SBMG). The surface condition evolutions before and after thermal exposure at 700 °C for 24 h were investigated. The fatigue performances of specimens after thermal exposure were evaluated. The results show that the surface roughness Ra after SB, SBSP and SBMG processes were 3.14, 2.35 and 0.04 µm, respectively. After thermal exposure, they almost remained unchanged for all three processes. The SB process caused work hardening in the near-surface region and the work hardening reached saturation after the SB process. Due to the mechanical grinding (MG) process removing the uncertain thick hardening layer, the maximum hardness after SBMG process was noticeably lower than those after SB and SBSP processes. After thermal exposure, the maximum hardness after SB, SBSP and SBMG processes significantly recovered. The SBMG specimens had the highest fatigue limit of 350 MPa. This is attributed to the SBMG specimens having very smooth surfaces and some work hardening remaining near their surface layers.

## 1. Introduction

Titanium aluminide (TiAl) alloys have received considerable attention as some of the most promising light-structural materials for aerospace applications, such as high-pressure compressors and low-pressure turbine blades, due to their low densities, high specific strengths and relatively high service temperatures of up to 800 °C [1,2,3,4,5]. TiAl alloy blades are subjected to the high frequency vibrations in service resulting from take-off, flight and landings of airplane. It has previously been observed that 80–90% of the dominant cracks initiated at the surface in TiAl_47_Cr_2_Nb_2_ (at.%) alloy fatigue specimens [6].

Over the past three decades, scholars have carried out some studies on the surface conditions of TiAl alloys produced by different machining methods, such as turning, milling, electro-discharge machining (EDM) and laser cutting; and produced by different surface processes, such as shot-peening (SP) and electropolishing. Meanwhile, the effects of different surface conditions on fatigue performance have been investigated. Trail et al. [7] found that residual tensile stress and microcracking were introduced by EDM, and the fatigue lives of EDM specimens were obviously reduced compared with polished specimens. Mantle et al. [8] found that deformed lamellae/surface drag, material pull-out/cracking and increased microhardness were formed at the surface layer after single-point turning. However, the differences in S–N fatigue curves between turned and polished specimens were minimal, both having an endurance limit of approximately 350 MPa. Bentley et al. [9] found that the high hardness and plastic deformation of the lamellae existed at the surface layer after high-speed milling, and the high-speed milling significantly increased fatigue strength by as much as 200 MPa over polished specimens. Yao et al. [10] investigated the effects of tool and turning parameters on surface integrity and fatigue behavior on turning the γ-TiAl alloy. They found that the fatigue life of the turning-polishing specimen with arithmetical mean deviation of the profile Ra 0.15 µm increased three times from that of the turning specimen with Ra 0.43 µm. Huang et al. [11,12,13,14,15] made great efforts to investigate the fatigue responses of different TiAl alloys with varied surface conditions and thermal exposure history. They found that the specific fatigue behavior should be discussed individually based upon the combined changes induced by surface conditions and thermal exposure history.

Although the relationships between surface conditions and fatigue performances in TiAl alloys have been preliminarily established, little work has been done to reveal the relationship between investment casting surface condition and fatigue performance. The fatigue specimens are generally taken out of TiAl ingots in the central area for most scholars. There has been little work done to study the surface conditions and fatigue performances of investment casting specimens. Investment casting is the most effective way to produce TiAl_47_Cr_2_Nb_2_ components with complicated shapes. After pouring and solidification, shell removal is performed to obtain the investment castings. Before delivery, the investment castings have to go through a sand-blasting (SB) process to remove the residual shell and obtain a good surface finish. Some investment castings with SB surface condition are used directly, whereas the others are further processed by shot-peening or mechanical grinding (MG) to improve the fatigue strength and damage tolerance. In view of this situation, more efforts should be made to investigate the surface condition evolutions and evaluate the fatigue performances of TiAl investment castings.

In this paper, three kinds of surface processes, namely, sand-blasting (SB); sand-blasting and shot-peening (SBSP); and sand-blasting and mechanical grinding (SBMG), were applied to the investment casted TiAl fatigue specimens. The three surface conditions were characterized in detail by using a roughness measuring instrument, scanning electron microscopy, a 3D non-contact optical profiler and a microhardness tester. Then, the fatigue performances of specimens with different surface conditions were evaluated. This study is helpful for understanding the fatigue failure of the TiAl alloys with different surface conditions.

## 2. Materials and Methods

### 2.1. Material and Heat Treatment

**Figure 1:**
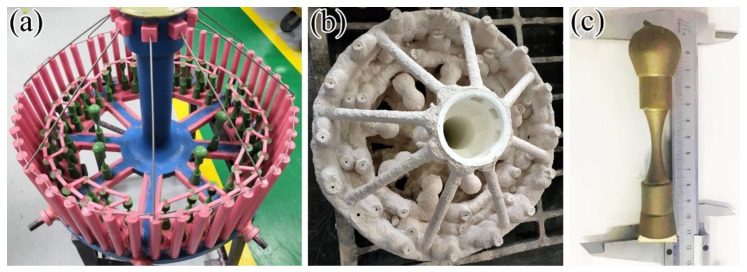
Investment casting system and fatigue specimen: (**a**) wax mold; (**b**) ceramic mold; (**c**) fatigue specimen.

A TiAl ingot with the nominal composition of TiAl_47_Cr_2_Nb_2_ was first prepared by vacuum arc melting, then vacuum induction remelting and finally, vacuum arc re-melting. Then, the ingot was placed into a vacuum arc furnace for remelting and poured into an Y_2_O_3_ coated ceramic mold preheated to 800 °C to produce the investment casted fatigue specimens. Figure 1a,b shows the wax mold and ceramic mold of the investment casting system, respectively. Figure 1c shows the investment casting fatigue specimen cut off from the investment casting system. The chemical compositions of fatigue specimens are shown in Table 1. The Al, Cr and Nb contents were determined using the chemical titration method. The O content was detected using the infrared absorption spectrometry. After casting, the fatigue specimens were first subjected to a hot isostatic press (HIP) process under an argon pressure of 172 MPa at 1260 °C for 4 h to eliminate casting defects. Then, they were isothermally held at 1185 °C for 6 h in a vacuum furnace, followed by furnace cooling (FC) to room temperature. This whole process is based on the investment casting technology for production of TiAl low pressure turbine blades [16].

### 2.2. Surface Process and Thermal Exposure Experiments

Three kinds of surface processes, namely, SB, SBSP and SBMG, were applied to the sheet specimens and fatigue specimens. The sheet specimens with dimensions of 10 × 10 × 8 mm^3^ were electro-discharge machined from the gating system of the casting, and the square surfaces were subjected to surface processes. The sheet specimens were used to study the surface condition evolutions after different surface processes.

The SB process was carried out manually using an inject type system. The corundum spheres with an average diameter of 0.5 mm were ejected at an air-jet pressure of 0.6 MPa in the air chamber. The SP process was performed with a numerically controlled machine tool, also using an inject type system. The zirconia-based ceramic shots with an average diameter of 0.3 mm were ejected at an air-jet pressure of 0.1 MPa in an air chamber. The angle between spray gun and specimen surface was controlled at close to 90°. The SP intensity was 0.3 mm, measured by the N-type Almen test piece. All peening procedures were performed to achieve full coverage. The MG process was performed using the sandpapers from 260 grit to 2000 grit to grind the surfaces of specimens, and the MG direction was parallel to the axis of the specimens.

After surface processes, the thermal exposure at 700 °C in an air-circulated furnace was applied to the specimens, including the sheet specimens and fatigue specimens. The temperature of thermal exposure corresponds to the anticipated service conditions of the TiAl_47_Cr_2_Nb_2_ alloy. They were kept at temperature for 24 h, then cooled to room temperature in the furnace.

### 2.3. Material Characterization

The surface topographies were characterized via scanning electron microscopy (SEM) in the secondary electron (SE) mode and 3D non-contact optical profiler LEXT OLS4000 (Tokyo, Japan). The surface roughness was measured using a TA620 (Beijing, China) roughness measuring instrument. The sampling length for measurement was 0.8 mm, and the assessment length was 5.0 mm. The arithmetical mean deviation of the profile, namely, Ra, was measured five times for each surface process. Microhardness profiles were measured to characterize work hardening and residual compressive stresses in the near-surface regions after three processes. In order to get as many indentations as possible in the near-surface regions, the measurements were conducted on a microhardness tester 402MVD (Lakebrav, IL, USA) with a lower load of 25 g and holding time of 6 s. The direction of measurement was perpendicular to the processed surface, and the first indentation was 10 µm from the processed surface. The microstructure was characterized via SEM in the backscattered electron (BSE) mode. The specimens for SEM-BSE were cut firstly, then carefully ground and polished using nano-silica slurry.

### 2.4. Mechanical Tests

**Figure 2:**
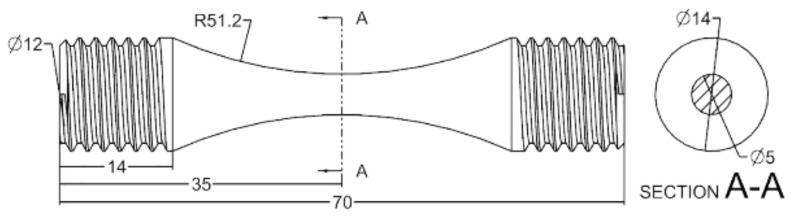
Dimensions of the fatigue specimens (unit: mm).

The S–N fatigue tests were conducted on a high-frequency fatigue testing machine, QBG-100, in a load-controlled mode at room temperature. The dimensions of the fatigue specimens are shown in Figure 2. The specimens were axially loaded in sinusoidal waveform with stress ratio R (R = σ_min_/σ_max_, where the σ_min_ and σ_max_ represent the minimum and maximum stresses applied to the fatigue specimens) of 0.1 and frequency of 100 Hz. For each surface process, the fatigue tests were performed for up to 1 × 10^7^ cycles. The fatigue limit σ_FL_ is typically defined as the value of σ_max_ under which the fatigue specimen did not fail after 1 × 10^7^ cycles. If some of the specimens failed within 1 × 10^7^ cycles, a lower value was used for defining the fatigue limit.

## 3. Results and Discussion

### 3.1. Microstructure of the Alloy

**Figure 3:**
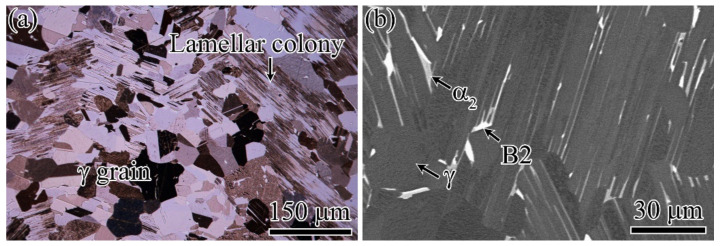
Optical (**a**) and SEM-BSE (**b**) microstructures of TiAl_47_Cr_2_Nb_2_ after heat treatment.

The microstructure of TiAl_47_Cr_2_Nb_2_ after heat treatment at 1185 °C exhibited a typical duplex structure, as shown in Figure 3a. The volume fractions of equiaxed γ grains and lamellar colonies were 55% and 45%, respectively. Figure 3b shows the SEM-BSE microstructure. The microstructure consisted of three phases, namely, γ phase (L1_0_ structure), α_2_ phase (D0_19_ structure) and B2 phase (CsCl structure), which are indicated as the dark, slightly bright and bright contrasts, respectively. The equiaxed grain region was mainly composed of γ phase and mixed with small amounts of α_2_ and B2 phases. Many α_2_ lamellae in lamellar colonies partly dissolved and transformed into γ phase, resulting in the coarsening of γ lamellae.

### 3.2. Surface Condition Characterization

#### 3.2.1. Surface Roughness

The surface roughness, Ra, is listed in Table 2 after each surface process, with an error range assessed by standard deviation. Before thermal exposure, the surface roughness values after SB, SBSP and SBMG processes were 3.14, 2.35 and 0.04 µm, respectively. The minimum surface roughness was produced by the SBMG process; the maximum surface roughness was produced by the SB process; and the middle one was produced by the SBSP process. Compared with the SB process, subsequent SP and MG processes can reduce the roughness of the SB surface. However, the surface roughness after the SBSP process was still large, and the surface roughness after the SBMG process showed a significant decrease. After thermal exposure at 700 °C for 24 h, the Ra increased slightly or almost remained unchanged for each surface process compared with that before thermal exposure.

#### 3.2.2. Surface Topography

**Figure 4:**
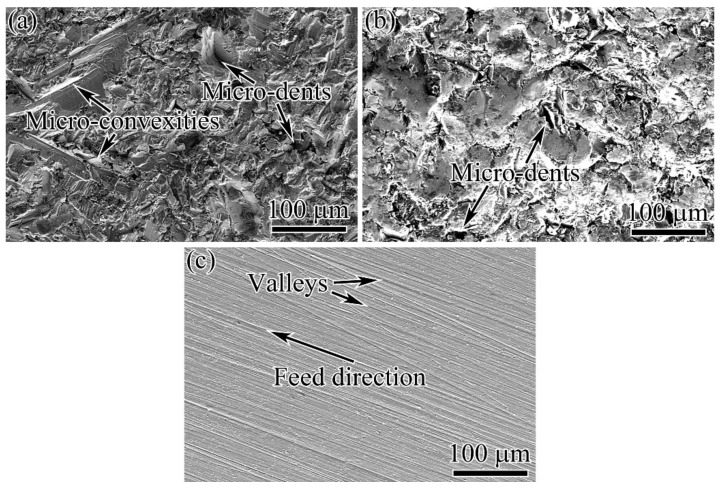
Surface morphologies after different surface processes before thermal exposure: (**a**) SB; (**b**) SBSP; (**c**) SBMG.

The surface morphologies after different surface processes before thermal exposure are shown in Figure 4. The SB process produced a rough surface, at which lots of micro-convexities and micro-dents were unevenly distributed, as shown in Figure 4a. SP of the SB surface removed most of the sharper micro-convexities and left surface residual height smaller than that on the SB surface, as presented in Figure 4b. MG carried out on the SB surface produced a very smooth surface, just leaving some extremely shallow valleys and a few small micro-dents on the surface, as shown in Figure 4c. These valleys resulted from the relative movement between the specimen surface and sandpaper, and they had a direction parallel to the feed direction. The small micro-dents were derived from the deep micro-dents on SB surface that cannot be completely removed by MG process.

**Figure 5:**
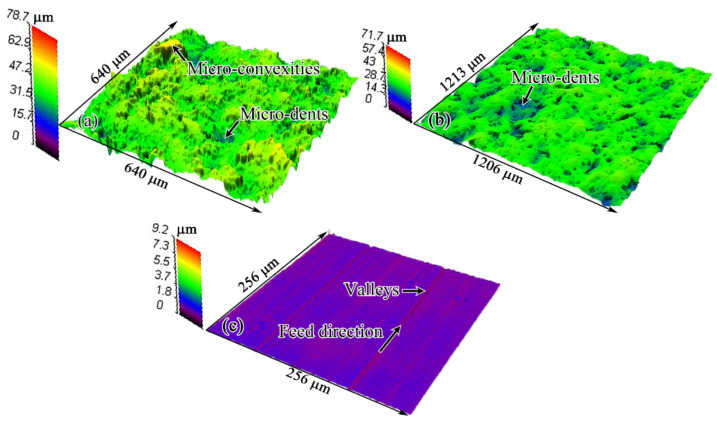
Surface topographies and roughness after different surface processes before thermal exposure: (**a**) SB; (**b**) SBSP; (**c**) SBMG.

**Figure 6:**
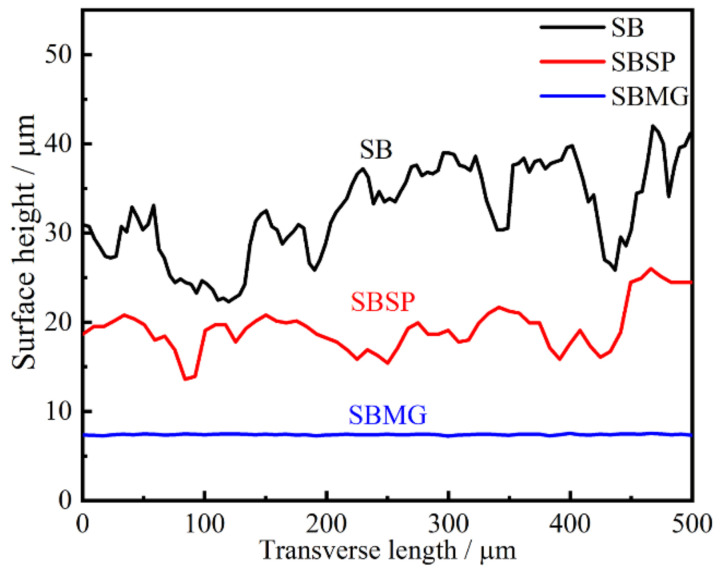
Surface profiles after different surface processes before thermal exposure.

The surface topographies after different surface processes before thermal exposure are shown in Figure 5. Characteristics of surfaces, including surface finish and surface fluctuation, can be observed more clearly in this way. The distribution of micro-convexities on the SB surface was extremely uneven. Especially for the sharp micro-convexities, they got together and were located in some areas on the SB surface, as presented in Figure 5a. After the SBSP process, no clear micro-convexities could be observed at the surface, as shown in Figure 5b. Additionally, the distribution of micro-dents seemed to be more uniform. The SBMG surface showed an extremely high surface finish, as presented in Figure 5c. Figure 6 shows the surface profiles of the specimens subjected to different surface processes. Due to the SB process being performed manually, the duration and distance from nozzle to specimen for each zone on the surface could not be controlled precisely. In this case, the SB surface profile had the largest fluctuation and Ra. Differently from the SB process, the SP process was conducted on the numerically controlled machine tool. The duration and distance from nozzle to specimen could be precisely kept at a constant value for the whole surface. In view of this situation, the SBSP surface profile had the middle fluctuation and Ra. The MG process can remove almost all micro-convexities and micro-dents, and only some extremely shallow valleys and a few smaller micro-dents remained on the surface. Thus, the SBMG surface profile was almost a straight line and had the lowest Ra.

**Figure 7:**
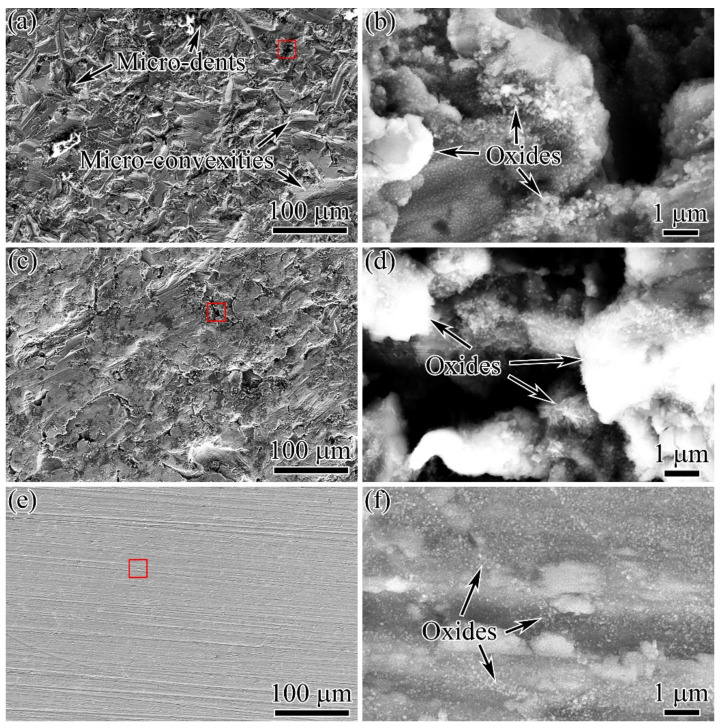
Surface morphologies (**a**,**c**,**e**) and enlarged views of the areas marked by the red rectangular wire frames (**b**,**d**,**f**) after different surface processes followed by thermal exposure: (**a**,**b**) SB; (**c**,**d**) SBSP; (**e**,**f**) SBMG.

The surface morphologies after different surface processes, followed by thermal exposure at 700 °C for 24 h, are shown in Figure 7. By comparing Figure 4a with Figure 7a; Figure 4b with Figure 7c; and Figure 4c with Figure 7e, it could be found that the surface morphologies before and after thermal exposure were almost the same. Figure 7b,d,f shows the enlarged views of the areas marked by the red rectangular wire frames in Figure 7a,c,e, respectively. The oxidation behaviors were observed on each surface. Some oxides exhibited a pointed shape, but a small number of oxides exhibited a needle shape. The oxides were generally mixed corundum α-Al_2_O_3_ and rutile TiO_2_ [17]. The oxides were very small, and the distribution of oxides was sparse. Huang’s research indicated that long-term oxidation for up to 10,000 h at 700 °C caused a noticeable increase in surface roughness after each surface process [11]. This indicates that TiAl alloys exhibit superior oxidation resistance at 700 °C for moderate durations. No obvious changes in Ra after thermal exposure can be attributed to the slight oxidation at 700 °C.

#### 3.2.3. Microhardness

**Figure 8:**
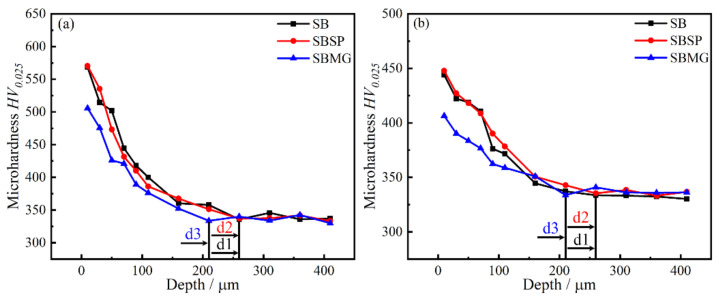
Microhardness distributions after different surface processes: (**a**) before thermal exposure; (**b**) after thermal exposure.

Figure 8 shows the microhardness distributions after different surface processes. Due to the process-induced compressive stresses and plastic deformation, the hardness in near-surface region significantly increased with a maximum value at the surface [18]. The maximum hardness and analysis are listed in Table 3. Before thermal exposure, the maximum hardness (h_max_) values were 568 ± 7.7 HV_0.025_, 570 ± 4.1 HV_0.025_ and 505 ± 3.1 HV_0.025_ for SB, SBSP and SBMG processes, respectively. Then, the microhardness dropped gradually from the maximum value to the baseline level of 335 HV_0.025_. Compared with the baseline level, the increments were 69%, 70% and 50% for SB, SBSP and SBMG processes, respectively. The depth of the hardening layer is abbreviated as d, and they were 260 µm (d1), 260 µm (d2) and 210 µm (d3) for SB, SBSP and SBMG processes, respectively. After thermal exposure, the maximum hardness values were 444 ± 6.5 HV_0.025_, 448 ± 4.5 HV_0.025_ and 406 ± 2.6 HV_0.025_ for SB, SBSP and SBMG processes, respectively. Compared with the baseline level, the increments were 32%, 33% and 21% for SB, SBSP and SBMG processes, respectively. As for the depths of surface hardening layers, they were still 260 µm (d1), 260 µm (d2) and 210 µm (d3) for SB, SBSP and SBMG processes, respectively. However, the maximum hardness had a noticeable decrease compared with that before thermal exposure. The decrements of the maximum hardness were 21%, 21% and 19% for SB, SBSP and SBMG processes, respectively.

The results above show that the SB and SBSP processes have the same maximum hardness and depth of hardening layer. This indicates that the work hardening reached the saturation after the SB process, and the subsequent SP process could not further increase the maximum hardness and depth of hardening layer. A similar result was found in the literature [19], in which the maximum hardness after one laser shock peening was 398.5 HV_0.5_ and the maximum hardness after three laser shock peening was 403.2 HV_0.5_. This small increment is attributed to the anterior impact improving the strength of the material in surface, and the latter impact struggles to generate the hardening in the same degree. The maximum hardness and depth of hardening layer after SBMG process were lower than those after SB and SBSP processes, which we attribute to the MG process removing an uncertain thick hardening layer.

The work hardening achieved by the SB process is prone to thermal exposure, which leads to a significant reduction in microhardness when compared with that before thermal exposure. In two-phase alloys, a significant contribution to work hardening was derived from dislocation dipoles and debris, which trailed and terminated at jogs in ordinary screw dislocations [20]. Isothermal and isochronal annealing experiments have shown that the dipole and debris defects are unstable upon annealing at moderately high temperatures [21,22]. In situ heating experiments inside the electron microscope have confirmed that the debris defects are rapidly annealed out. Therefore, the hardness values in hardening layers decrease obviously after thermal exposure in the TiAl_47_Cr_2_Nb_2_ alloy. The work hardening has not been completely eliminated, which might be attributed to the stable dislocation structures remaining after thermal exposure.

#### 3.2.4. Structural Changes beneath the Processed Surface

**Figure 9:**
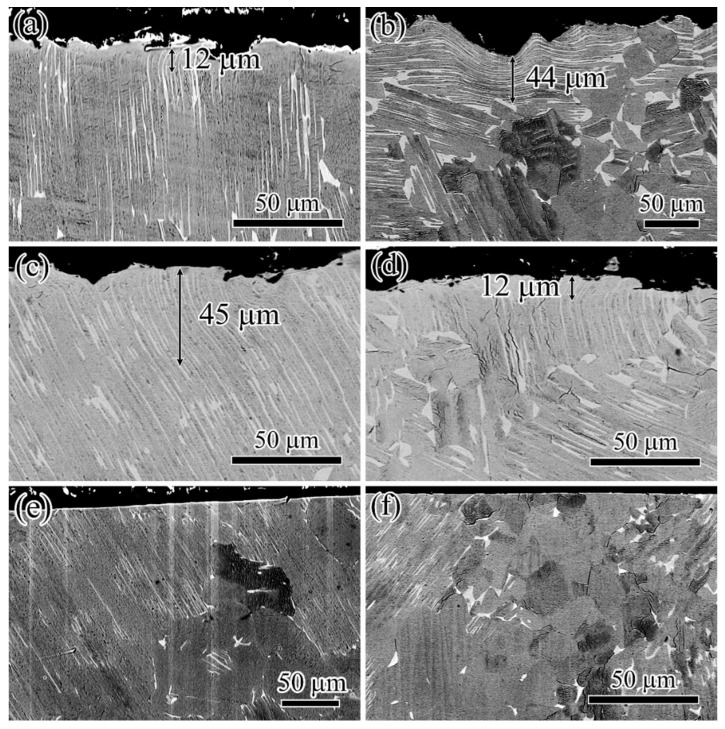
Cross-sectional micrographs for different surface processes before (**a**,**c**,**e**) and after (**b**,**d**,**f**) thermal exposure at 700 °C for 24 h: (**a**,**b**) SB; (**c**,**d**) SBSP; (**e**,**f**) SBMG.

The structural changes induced by different processes are demonstrated in Figure 9. Cross-sectional micrographs reveal the deformed layers existing on SB and SBSP surfaces, which are marked by double arrows in Figure 9a–d. The deformed layers were characterized by bending of both the α_2_ and γ lamellae. They were about 12–44 and 12–45 µm in depth for the SB surface and SBSP surface, respectively. This indicates that the high-stress layer induced by SB process was about 12–44 µm, which was significantly smaller than the depth of the hardening layer. To maintain the equilibrium of internal forces inside the specimen, the compensating compressive stresses should extend beyond the depth of the deformed layer [23]. As for the SBMG surface, there was no clear deformed layer (see Figure 9e,f) due to the MG process removing the high-stress layer induced by the SB process.

### 3.3. Fatigue Evaluation

#### 3.3.1. Fatigue Limit

**Figure 10:**
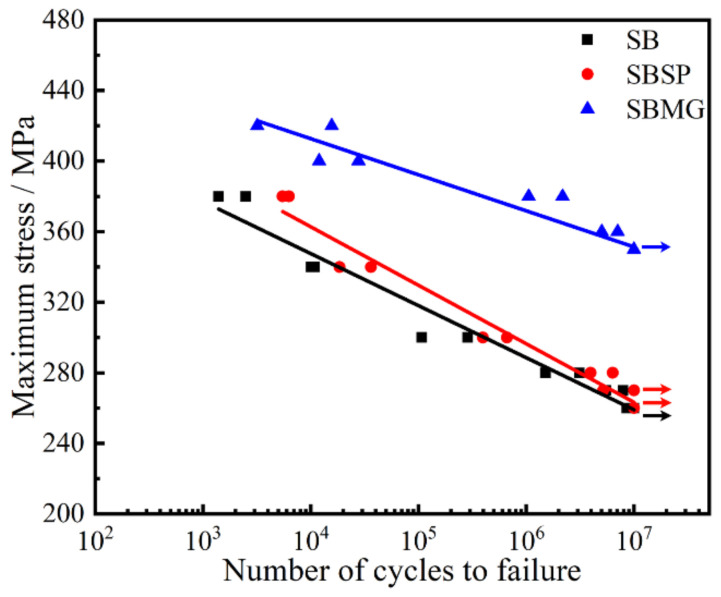
S–N curves for TiAl_47_Cr_2_Nb_2_ with different surface conditions.

The S–N curves and fatigue limits for TiAl_47_Cr_2_Nb_2_ with different surface conditions are shown in Figure 10 and Table 4, respectively. The results show that the surface condition has a great influence on the fatigue limit of TiAl_47_Cr_2_Nb_2_. As listed in Table 4, the fatigue limits were 260, 260 Pa and 350 MPa for SB, SBSP and SBMG surface conditions, respectively. Compared with that for the SB surface condition, the fatigue limit for the SBSP surface condition was not improved. However, the fatigue limit was significantly improved for the SBMG surface condition, and the increment reached 34%.

Surface roughness, work hardening and residual compressive stresses are the key factors that determine the fatigue limit of the material [24,25,26,27]. The enhanced surface roughness means many microcracks, micro-dents and overlaps on the surface, which will deteriorate the fatigue limit. Inversely, the enhanced work hardening and residual compressive stresses can offset or reduce applied tensile stresses, slow crack growth and inhibit or retard crack initiation, which will contribute to enhancing the fatigue limit. Therefore, when considering whether to improve the fatigue limit, the beneficial effects caused by work hardening and residual compressive stresses and the detrimental effects caused by surface roughness should be evaluated comprehensively.

Although the work hardening and residual compressive stresses of SB and SBSP specimens were higher than those of the SBMG specimens, the fatigue limit of SBMG specimens increased by 34% in comparison with those of SB and SBSP specimens. This must be attributed to the SBMG specimens having a very smooth surface with low surface roughness compared with SB and SBSP specimens with high surface roughness. The deep and sharp micro-dents on SB and SBSP surfaces can easily induce stress concentrations and contribute to crack initiation, thereby deteriorating the fatigue limit. Inversely, the SBMG surface had low surface roughness, and some work hardening and residual compressive stresses remained in the surface, which significantly improved the fatigue limit. Although the SP process reduced the surface roughness of the surface, the surface roughness of SBSP surface was still high, and the deep and sharp micro-dents still existed on the SBSP surface. Thus, the fatigue limit of the SBSP specimens was the same as that of the SB specimens. In fact, Figure 10 shows that a SBSP specimen did not fail after 1 × 10^7^ cycles under σ_max_ of 270 MPa, whereas all SB specimens failed before 1 × 10^7^ cycles under σ_max_ of 270 MPa. This indicates that the fatigue limit of SBSP specimens should be higher than that of SB specimens. However, the increment was very small due to the still high surface roughness of SBSP specimens.

#### 3.3.2. Fractography

**Figure 11:**
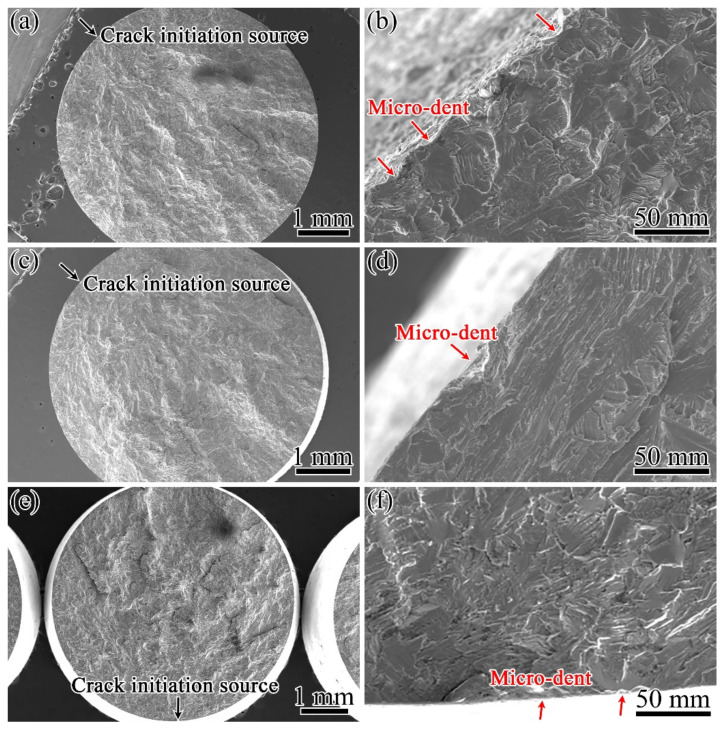
The whole fracture and crack initiation sources of fatigue specimens with different surface conditions: (**a**,**b**) SB; (**c**,**d**) SBSP; (**e**,**f**) SBMG.

Figure 11a,c,e presents the typical fractographs of the whole fractures of specimens with different surface conditions after fatigue tests. The fractures were mainly brittle, and there was no evidence of ductile fractures, such as dimples. The regions marked by the black arrows were magnified to further study the crack initiation, as shown in Figure 11b,d,f. All of the cracks initiated from the specimen surfaces where the micro-dents were located, as shown by the red arrows. These micro-dents can easily induce local stress concentrations under fatigue loading, and fatigue cracks initiate there and expand into the interiors of specimens.

**Figure 12:**
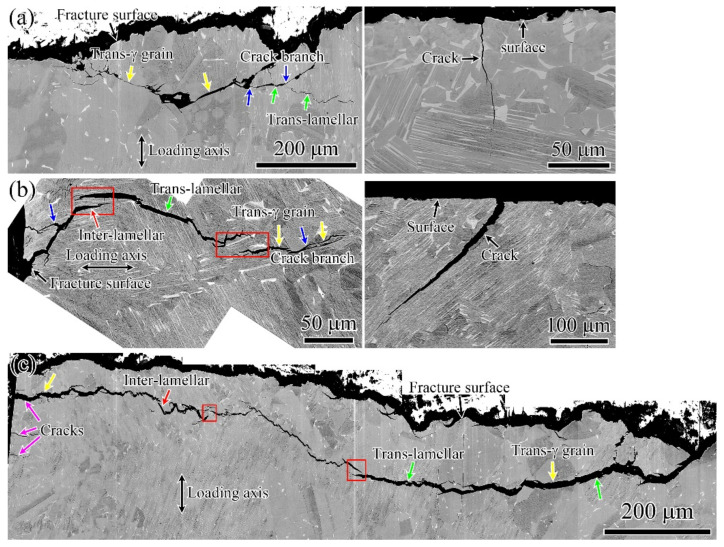
Crack propagation character of fatigue specimens with different surface conditions: (**a**) SB; (**b**) SBSP; (**c**) SBMG.

Figure 12 shows the longitudinal sections of fractures of fatigue specimens with different surface conditions. It reveals that the fatigue specimens had mainly trans-lamellar and trans-γ grain appearances, as indicated by the green and yellow arrows, respectively. A small number of inter-lamellar appearances are shown in Figure 12b,c, as shown by the red arrows. The kinking and branching of the cracks and crack-wake ligaments were formed during crack propagation, as presented by the blue arrows and red rectangular wire frames, respectively. Crack-tip measurements indicated that the crack-wake ligaments can reduce the near-tip stress intensity factor and improve fracture resistance if they remain intact during fatigue loading [28]. In addition, microcracks with a length of 27–269 µm were observed near the fracture surfaces, as shown in the right insets in Figure 12a–c shown by the pink arrows. These microcracks also initiated from the micro-dents, corresponding to the results shown in Figure 11b,d,f. Most of microcracks did not grow further into the critical length. The microcrack which grew into the critical length would transform into the main crack, and the crack propagation entered the steady stage. Then, the main crack continued to grow with the increase in fatigue cycles, and the crack propagation went into the accelerated propagation. Finally, the fatigue fracture happened. The multi-crack initiations may cause a decrease in the crack initiation resistance.

## 4. Conclusions

In this paper, the surface condition evolution and fatigue performance of TiAl_47_Cr_2_Nb_2_ after different surface processes were studied. The main results can be summarized as follows.

(1) The surface roughness (Ra) values after SB, SBSP and SBMG processes were 3.14, 2.35 and 0.04 µm, respectively. After thermal exposure at 700 °C for 24 h, they increased slightly or almost remained unchanged for all three processes.
(2) The SB process caused work hardening in near-surface region, and the work hardening reached saturation after the SB process. The maximum hardness and depth of hardening layer were nearly same for SB and SBSP processes, which were 570 HV_0.025_ and 260 µm, respectively. The maximum hardness and depth of hardening layer after SBMG process were 505 HV_0.025_ and 210 µm, respectively, which were noticeably lower than that after SB and SBSP processes. This was attributed to the MG process removing an uncertainly thick hardening layer.
(3) After thermal exposure at 700 °C for 24 h, the maximum hardness after SB, SBSP and SBMG processes significantly recovered to 444 HV_0.025_, 448 HV_0.025_ and 406 HV_0.025_, respectively. The depths of hardening layers remained unchanged.
(4) The SB and SBSP specimens had nearly same fatigue limit of 260 MPa. The fatigue limit of SBMG specimens was 350 MPa, increasing by 34% in comparison with those of SB and SBSP specimens. This was attributed to the SBMG specimens having a very smooth surface and keeping some work hardening near the surface layer.
(5) The fatigue fractures were typical brittle cleavage fractures. All of the cracks initiated from the specimen surfaces where the micro-dents were located. The fatigue specimens had trans-lamellar and trans-γ grain appearances.

## Figures and Tables

**Table 1:** Chemical composition of the TiAl_47_Cr_2_Nb_2_ alloy (at.%).

Ti	Al	Cr	Nb	O
Bal.	47.01	2.02	2.09	0.13

**Table 2:** Surface roughness Ra with error range after each surface process (unit: µm).

Surface Process	Before Thermal Exposure	After Thermal Exposure
SB	3.14 ± 0.05	3.28 ± 0.02
SBSP	2.35 ± 0.19	2.43 ± 0.17
SBMG	0.04 ± 0.003	0.10 ± 0.004

**Table 3:** Maximum hardness and analysis for different surface processes.

Thermal Exposure	Surface Process	h_max_/HV_0.025_	Change in h_max_ (%) Relative to Baseline Level	Change in h_max_ (%) Relative to No Thermal Exposure
Before	SB	568 ± 7.7	69	-
	SBSP	570 ± 4.1	70	-
	SBMG	505 ± 3.1	50	-
After	SB	444 ± 6.5	32	−21
	SBSP	448 ± 4.5	33	−21
	SBMG	406 ± 2.6	21	−19

**Table 4:** Fatigue limits and analysis for TiAl_47_Cr_2_Nb_2_ with different surface conditions.

Surface Condition	σ_FL_/MPa	Change in σ_FL_ (%) Relative to SB
SB	260	-
SBSP	260	0
SBMG	350	34

## Data Availability

Not applicable.

## References

[1] Sun Z., Zhu L., Mo X., Nan H., Ding X. (2021). Microstructure characterization and properties of graphene oxide-reinforced TiAl matrix composites. Metals.

[2] Chen G., Peng Y., Zheng G., Qi Z., Wang M., Yu H., Dong C., Liu C. (2016). Polysynthetic twinned TiAl single crystals for high-temperature applications. Nat. Mater..

[3] Wang Y., Lin J., He Y., Wang Y., Chen G. (2007). Microstructure and mechanical properties of as-cast Ti–45Al–8.5Nb–(W,B,Y) alloy with industrial scale. Mater. Sci. Eng. A.

[4] Appel F., Oehring M., Wagner R. (2000). Novel design concepts for gamma-base titanium aluminide alloys. Intermetallics.

[5] Voice W. (1999). The future use of gamma titanium aluminides by Rolls-Royce. Aircr. Eng. Aerosp. Technol..

[6] Jones P.E., Eylon D. (1999). Effects of conventional machining on high cycle fatigue behavior of the intermetallic alloy Ti-47Al-2Nb-2Cr (at.%). Mater. Sci. Eng. A.

[7] Trail S.J., Bowen P. (1995). Effects of stress concentrations on the fatigue life of a gamma-based titanium aluminide. Mater. Sci. Eng. A.

[8] Mantle A.L., Aspinwall D.K. (1997). Surface integrity and fatigue life of turned gamma titanium aluminide. J. Mater. Process. Technol..

[9] Bentley S.A., Mantle A.L., Aspinwall D.K. (1999). The effect of machining on the fatigue strength of a gamma titanium aluminide intertmetallic alloy. Intermetallics.

[10] Yao C., Lin J., Wu D., Ren J. (2018). Surface integrity and fatigue behavior when turning γ-TiAl alloy with optimized PVD-coated carbide inserts. Chin. J. Aeronaut..

[11] Huang Z., Lin J., Zhao Z., Sun H. (2017). Fatigue response of a grain refined TiAl alloy Ti-44Al-5Nb-1W-1B with varied surface quality and thermal exposure history. Intermetallics.

[12] Huang Z., Lin J., Sun H. (2017). Microstructural changes and mechanical behaviour of a near lamellar γ-TiAl alloy during long-term exposure at 700 °C. Intermetallics.

[13] Huang Z., Lin J., Feng B. (2017). Microstructural characterization and fatigue response of alloy Ti-46Al-5Nb-1W with varied surface quality and thermal exposure history. Mater. Charact..

[14] Huang Z., Huang S. (2015). On the role of thermal exposure on the stress controlled fatigue behaviour of an intermediate strength γ-TiAl based alloy. Mater. Sci. Eng. A.

[15] Huang Z., Sun C. (2014). On the role of thermal exposure on the stress controlled fatigue behaviour of a high strength titanium–aluminum alloy. Mater. Sci. Eng. A.

[16] Aguilar J., Schievenbusch A., Kättlitz O. (2011). Investment casting technology for production of TiAl low pressure turbine blades—Process engineering and parameter analysis. Intermetallics.

[17] Qu S., Tang S., Feng A., Feng C., Shen J., Chen D. (2018). Microstructural evolution and high-temperature oxidation mechanisms of a titanium aluminide based alloy. Acta Mater..

[18] Unal O., Cahit Karaoglanli A., Varol R., Kobayashi A. (2014). Microstructure evolution and mechanical behavior of severe shot peened commercially pure titanium. Vacuum.

[19] Nie X., He W., Zhou L., Li Q., Wang X. (2014). Experiment investigation of laser shock peening on TC6 titanium alloy to improve high cycle fatigue performance. Mater. Sci. Eng. A.

[20] Appel F., Wagner R. (1998). Microstructure and deformation of two-phase γ-titanium aluminides. Mater. Sci. Eng. R Rep..

[21] Paul J.D.H., Appel F. (2003). Work-hardening and recovery mechanisms in gamma-based titanium aluminides. Metall. Mater. Trans. A.

[22] Appel F., Sparka U., Wagner R. (1999). Work hardening and recovery of gamma base titanium aluminides. Intermetallics.

[23] Lindemann J., Buque C., Appel F. (2006). Effect of shot peening on fatigue performance of a lamellar titanium aluminide alloy. Acta Mater..

[24] Takahashi K., Sato E. (2010). Influence of Surface Treatments on Fatigue Strength of Ti6Al4V Alloy. Mater. Trans..

[25] Fernández Pariente I., Guagliano M. (2008). About the role of residual stresses and surface work hardening on fatigue ΔKth of a nitrided and shot peened low-alloy steel. Surf. Coat. Technol..

[26] Nishida S.I., Zhou C.L., Hattori N., Wang S.W. (2007). Fatigue strength improvement of notched structural steels with work hardening. Mater. Sci. Eng. A.

[27] Jiang X.P., Man C.S., Shepard M.J., Zhai T. (2007). Effects of shot-peening and re-shot-peening on four-point bend fatigue behavior of Ti–6Al–4V. Mater. Sci. Eng. A.

[28] Chan K.S., Shih D.S. (1998). Fundamental aspects of fatigue and fracture in a TiAl sheet alloy. Metall. Mater. Trans. A.

